# HBXIP activates the PPARδ/NF-κB feedback loop resulting in cell proliferation

**DOI:** 10.18632/oncotarget.23057

**Published:** 2017-12-08

**Authors:** Qian Liu, Wenbin Lu, Chunxia Yang, Yue Wang, Wenjing Li, Ying Chu, Jianzhong Deng, Yongzhong Hou, Jianhua Jin

**Affiliations:** ^1^ Department of Oncology, The Changzhou Wujin People’s Hospital, Jiangsu Province, 213017, China; ^2^ Institute of Life Sciences, Jiangsu University, Zhenjiang, Jiangsu Province, 212013, China

**Keywords:** HBXIP, PPARδ, NF-κB, proliferation, colonic cancer

## Abstract

Hepatitis B X-interacting protein (HBXIP, also termed as LAMTOR5) plays a crucial role in regulation of cancer progression, while the mechanism is still unclear. Here we found that HBXIP increased the expression of PPARδ (peroxisome proliferator-activated receptor-δ) in gene and protein levels of SW480 or HT-29 colonic cancer cells. Chromatin immunoprecipitation and luciferase reporter assays showed that HBXIP occupied the core promoter (−1079/−239 nt) regions of PPARδ and that HBXIP activated the transcription activity of PPARδ in an NF-κB (p65)-dependent manner. Moreover, Co-immunoprecipitation and immunofluorescence analysis showed that HBXIP bound to NF-κB/p65 in the cells. Interestingly, we found that PPARδ could conversely increase the expression of NF-κB/p65 through activating its transcription activity. In addition, the clinical observations showed that both HBXIP and PPARδ were highly expressed in colonic carcinoma, and HBXIP expression was positively associated with that of PPARδ in the clinical specimen. Importantly, HBXIP expression levels were positively correlated with the clinical pathological parameters including lymph node metastasis and advanced TNM stage. These findings suggest that HBXIP served as a co-activator to activate the positive feedback regulations of NF-κB/PPARδ, which promoted the fast proliferation of the colonic cancer cells. Therapeutically, HBXIP may serve as a potential drug target of colonic cancer cells.

## INTRODUCTION

Colonic cancer is the third death-related cancer in the worldwide, accounting for more than 1,300,000 new cases annually and its incidence has sharply increased over the past two decades [1]. Hepatitis B X-interacting protein (HBXIP), a new oncoprotein also known as LAMTOR5 [2], is a conserved ～18 KDa protein originally identified by its interaction with the hepatitis B virus X protein [3]. Previous studies have reported that HBXIP functions as a coactivator of multiple oncogenic transcription factors, such as TF-IID, SP1, STAT3 on the promotion of proliferation and metastasis of breast cancer cells [4–6]. However, the mechanism by which HBXIP enhances the growth of colonic cancer remains poorly documented.

Peroxisome proliferator-activated receptors (PPARs) are a nuclear receptor family of ligand-inducible transcription factors, which have three isoforms: PPARα, δ and γ, which are expressed in all cell types of the brain [7, 8]. Many researchers have investigated the neuroprotective properties of PPAR agonists [9–11]. The efficiency of PPARδ agonists in neurodegenerative disease animal models have been reviewed [12, 13]. To date, studies demonstrate that PPARδ also play crucial role in the regulation of cell growth and metabolism of glucose and lipids [14–16]. PPARδ is highly expressed in colonic cells and implicated in colonic tumorigenesis, and its expression is elevated in human colorectal carcinoma specimen [17]. PPARδ also enhances the expression of VEGF, an angiogenic factor, in colonic carcinoma cells [18]. Moreover, the activation of PPARδ is associated with oncogenic pathways such as the K-Ras and APC/β-catenin/Tcf pathway [19]. PPARδ deficiency disrupts hypoxia-mediated tumorigenic potential of colonic cancer cells [20]. Additionally, PPARδ is required for chronic colonic inflammation and colitis-associated carcinogenesis [21].

In the present study, we investigated the mechanism by which HBXIP promoted the proliferation of colonic cancer cells. Our data indicate that the oncoprotein HBXIP, functioned as a co-activator of NF-κB (p65) or PPARδ, transactivated the promoter activity of PPARδ or NF-κB, and enhanced the expression of PPARδ and NF-κB in mRNA and protein levels in a positive feedback loop manner, leading to the fast growth of colonic cancer cells. These findings contribute new insight into the mechanism by which HBXIP enhances the proliferation of colonic cancer cells, and also provide a potential target for cancer treatment.

## RESULTS

### HBXIP up-regulates PPARδ expression in colonic cancer cells

**Figure 1:**
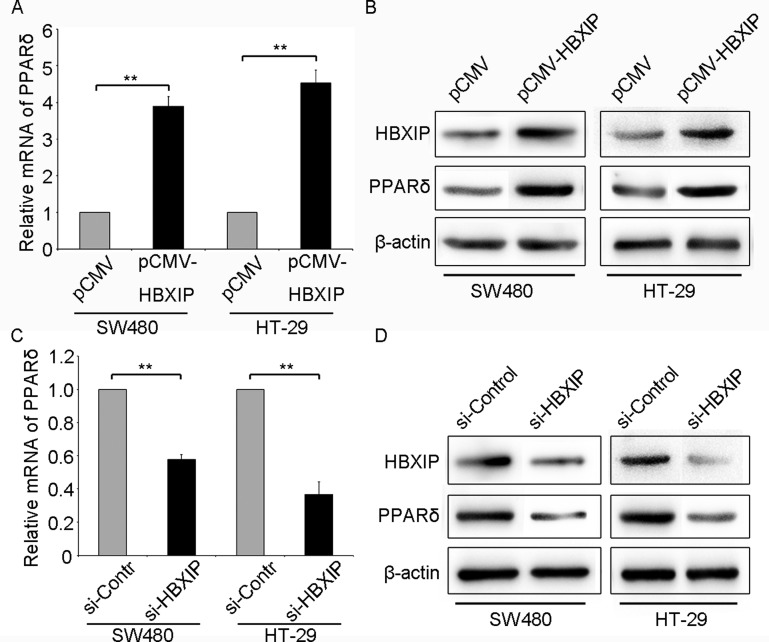
HBXIP up-regulates PPARδ expression in colonic cancer cells (**A**) The mRNA or protein (**B**) levels of PPARδ were tested by qRT-PCR or Western blot analysis in SW480 and HT-29 cells transiently transfected with pCMV (1 μg) or pCMV-HBXIP (1 μg), respectively. The mRNA (**C**) and protein (**D**) levels of PPARδ were detected by qRT-PCR or Western blot in SW480 or HT-29 cells transiently transfected with negative control siRNA (si-Control, 100 nM) or HBXIP siRNA (si-HBXIP, 100 nM), respectively.

Studies had demonstrated that HBXIP (LAMTOR5) expression was up-regulated in many types of cancers [4, 22, 23]. And PPARδ is highly expressed in colonic cells and implicated in colonic tumorigenesis [17]. Then we wondered whether the expression of HBXIP was associated with that of PPARδ in the colonic cancer cells. Intriguingly, we observed that the mRNA and protein levels of PPARδ were significantly elevated by transiently transfecting HBXIP expression plasmids (pCMV-HBXIP) in colonic cancer SW480 and HT-29 cells (Figure 1A, 1B and Supplementary Figure 1B). Conversely, the expression of PPARδ was remarkably reduced when HBXIP was knockdown by transiently transfecting HBXIP siRNA (si-HBXIP) in colonic cancer SW480 or HT-29 cells respectively (Figure 1C and 1D). Therefore, we conclude that the oncoprotein HBXIP can up-regulate the expression of PPARδ in colonic cancer cells.

### HBXIP promotes PPARδ gene transcription activity

**Figure 2:**
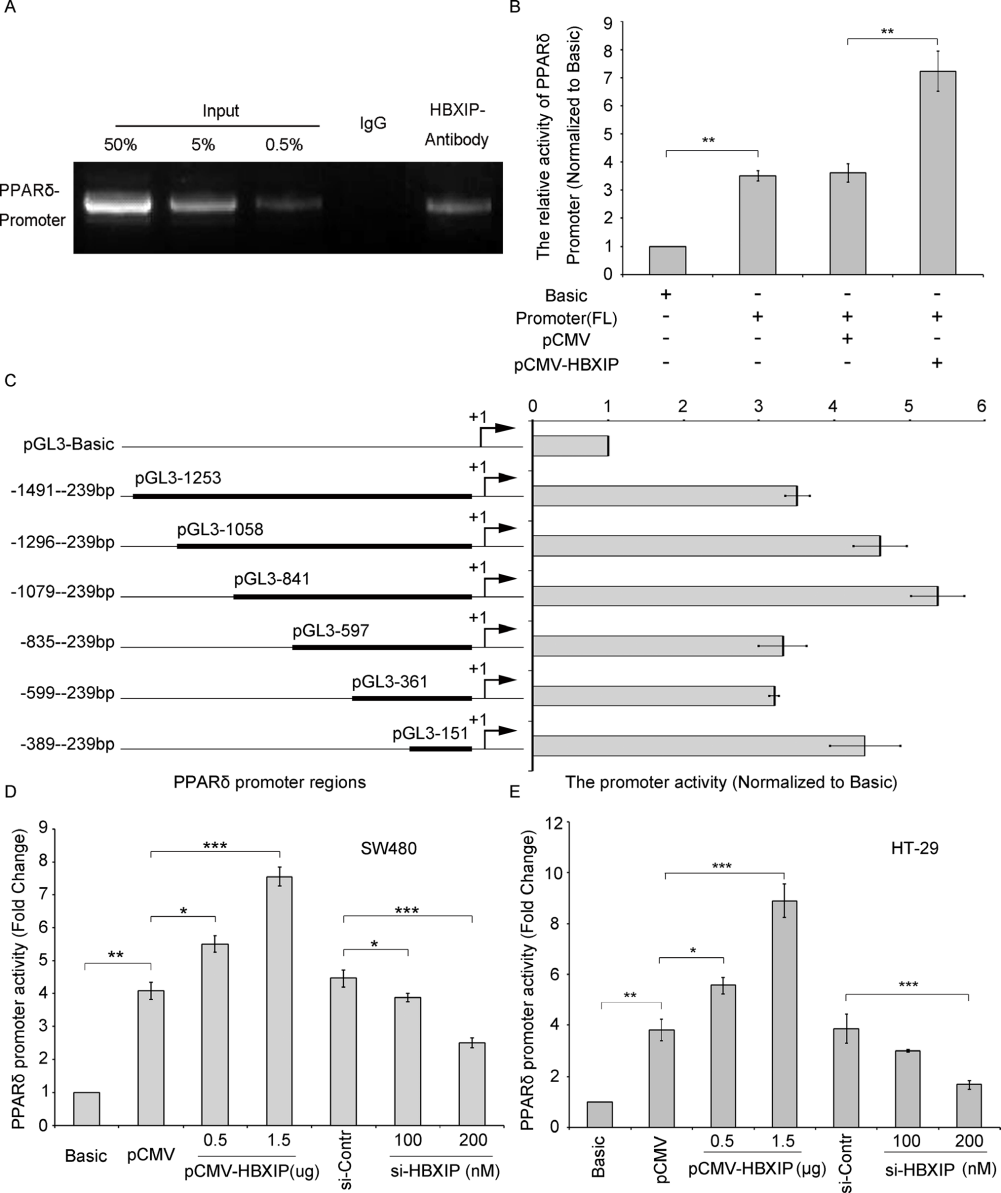
HBXIP is able to activate PPARδ transcription activity (**A**) The interaction between HBXIP and PPARδ promoter was detected by ChIP assay. The ChIP products were validated by sequencing and the data presented are from three independent experiments. (**B**) The promoter activity of PPARδ was tested by dual luciferase reporter system in SW480 cells transiently transfected with pCMV (0.5 μg) or pCMV-HBXIP (0.5 μg). (**C**) The relative activities of different promoter regions of PPARδ were examined by dual luciferase reporter system respectively. (**D** and **E**) The relative activities of the core promoter (−1079/−239 nt) of PPARδ were measured by dual luciferase reporter system in SW480 or HT-29 cells transiently transfected with pCMV-HBXIP (0.5 and 1.5 μg) or si-HBXIP (100 or 200 nM), respectively. *p < 0.05, **p < 0.01, ***p < 0.001. Student’s *t*-test. All experiments were performed three times.

Our previous report indicated that HBXIP could function as a co-activator of TFIID to activate the Lin28B transcription activities [4]. Accordingly, we wondered whether HBXIP could be involved in the transcriptional regulation of PPARδ. As expected, HBXIP could occupy the PPARδ promoter regions by chromatin immunoprecipitation (ChIP) assays (Figure 2A). Then we constructed the PPARδ promoter (−1491/−239 nucleotides region) into pGL3-Basic vector and luciferase reporter gene assays were performed, the data showed that the activity of PPARδ promoter could be markedly enhanced by HBXIP in SW480 cells (Figure 2B), suggesting that HBXIP up-regulates PPARδ expression *via* activating PPARδ promoter. Next, we further identified the PPARδ promoter core region. Various lengths of the PPARδ 5′-flanking regions, including -1491/-239 (pGL3-1253), -1296/-239 (pGL3-1058), -1079/-239 (pGL3-841), -835/-239 (pGL3-597), -599/-239 (pGL3-361) and -389/-239 (pGL3-151), were cloned and transiently transfected into SW480 cells to measure promoter activities respectively. The luciferase reporter gene assays were performed and the results indicated that pGL3-841 exhibited the maximum promoter activity among these promoter regions (Figure 2C), indicating that the region of -1079/-239 nt is the promoter core region of PPARδ. Co-transfections of pCMV-HBXIP or si-HBXIP with pGL3-841 or pGL3-Basic control were performed in SW480 and HT-29 cells respectively. The results demonstrated that the promoter activities of pGL3-841 increased by 4- or 6-fold in the HBXIP-transfected SW480 or HT-29 cells respectively (Figure 2D and 2E). Meanwhile, the promoter activities of pGL3-841 decreased by 2- or 3-fold in 200 nM/well si-HBXIP-transfected SW480 or HT-29 cells (Figure 2D and 2E). Thus, These results suggest that HBXIP up-regulted the gene expression of PPARδ through binding and activating the core promoter (−1079/−239 nt) of PPARδ.

### HBXIP activates PPARδ promoter in an NF-κB-dependent manner

**Figure 3:**
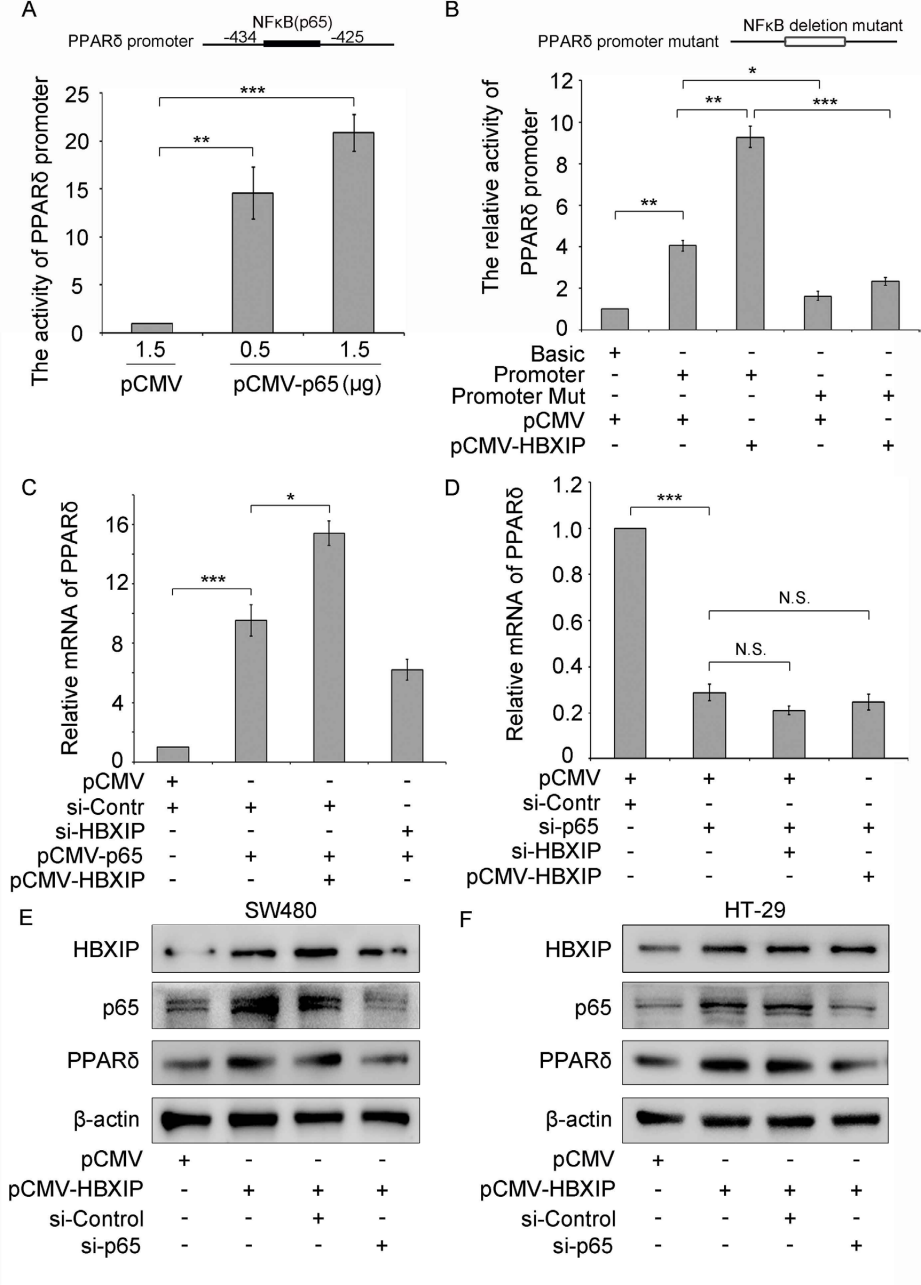
NF-κB is required for PPARδ transcription activation (**A**) The promoter activities of PPARδ were tested by dual luciferase reporter system in SW480 cells transiently transfected with pCMV (1.5 μg) or pCMV-p65 (0.5 or 1.5 μg), respectively. (**B**) The relative activities of PPARδ promoter and promoter mutant (NF-κB binding sequence deletion mutant) were detected by dual luciferase reporter system in SW480 cells transiently transfected with pCMV (1 μg) or pCMV-HBXIP (1 μg). (**C** and **D**) The mRNA levels of PPARδ were detected by qRT-PCR in SW480 cells transiently cotransfected with pCMV/si-Control, si-Control/pCMV-p65, si-HBXIP/pCMV-p65 si-Control/pCMV-HBXIP/pCMV-HBXIP, pCMV/si-p65, pCMV/si-p65/si-HBXIP and si-p65/pCMV-HBXIP, respectively. (**E** and **F**) The expression levels of p65 and PPARδ were tested by Western blot in SW480 or HT-29 cells transiently transfected with pCMV (1 μg), pCMV-HBXIP (1 μg), pCMV-HBXIP (1 μg)/si-Control (100 nM) and pCMV-HBXIP (1 μg)/si-p65 (100 nM), respectively.

We next explored the -1079/-239 nt promoter regions for a possible transcription factor binding sites by using GPMiner (http://gpminer.mbc.nctu.edu.tw/). The promoter region −1079/−239 contains various different promoter elements, such as Hsf1 and NF-κB/p65. It has been reported that NF-κB/p65 signaling was involved in carcinogenesis [24]. Thus, we hypothesized that HBXIP might activate the PPARδ promoter *via* NF-κB/p65. Interestingly, luciferase reporter gene assays indicated that PPARδ promoter activity was remarkably increased in a dose-dependent manner in SW480 cells treated with p65 expression plasmid (pCMV-p65) (Figure 3A), suggesting that NF-κB can activate the transcription activity of PPARδ. To further validate whether NF-κB is an important transcription factor of PPARδ promoter, we reconstructed the pGL3-841 mutant, which deleted the NF-κB binding sequence (PPARδ promoter mutant), and performed the luciferase reporter gene assays. Data showed that the activity of pGL3-841 mutant sharply decreased compared to that of the normal promoter. Moreover, HBXIP failed to activate the pGL3-841 mutant (Figure 3B), indicating that HBXIP activates the PPARδ promoter activity in an NF-κB-dependent manner. To further verify that HBXIP activates PPARδ transcription activity *via* NF-κB, the quantitative real-time PCR was performed and the results showed that the mRNA levels of PPARδ of SW480 cells co-transfected with pCMV-p65/pCMV-HBXIP markedly increased compare to that of the cells transfected with pCMV-p65. The mRNA levels of PPARδ of cells co-transfected with pCMV-p65/ si-HBXIP decreased compared to that of cells transfected with pCMV-p65 (Figure 3C and Supplementary Figure 2A), indicating that HBXIP up-regulates PPARδ expression *via* NF-κB. Moreover, the mRNA levels of PPARδ of SW480 cells transfected with si-p65 decreased relative to that of cells transfected with si-Control, and HBXIP failed to enhance the PPARδ mRNA levels in the cells treated with transfecting si-p65 (Figure 3D), indicating that HBXIP up-regulated PPARδ expression in a NF-κB-dependent manner. We then detected the protein levels of PPARδ by Western blotting. The results demonstrated that over-expression of HBXIP increased PPARδ expressions in SW480 or HT-29 cells. However, the knockdown of p65 abolished the increase of PPARδ proteins-induced by HBXIP (Figure 3E, 3F and Supplementary Figure 2B), indicating that HBXIP boosts the expression of PPARδ in a NF-κB-dependent manner in cancer cells.

**Figure 4:**
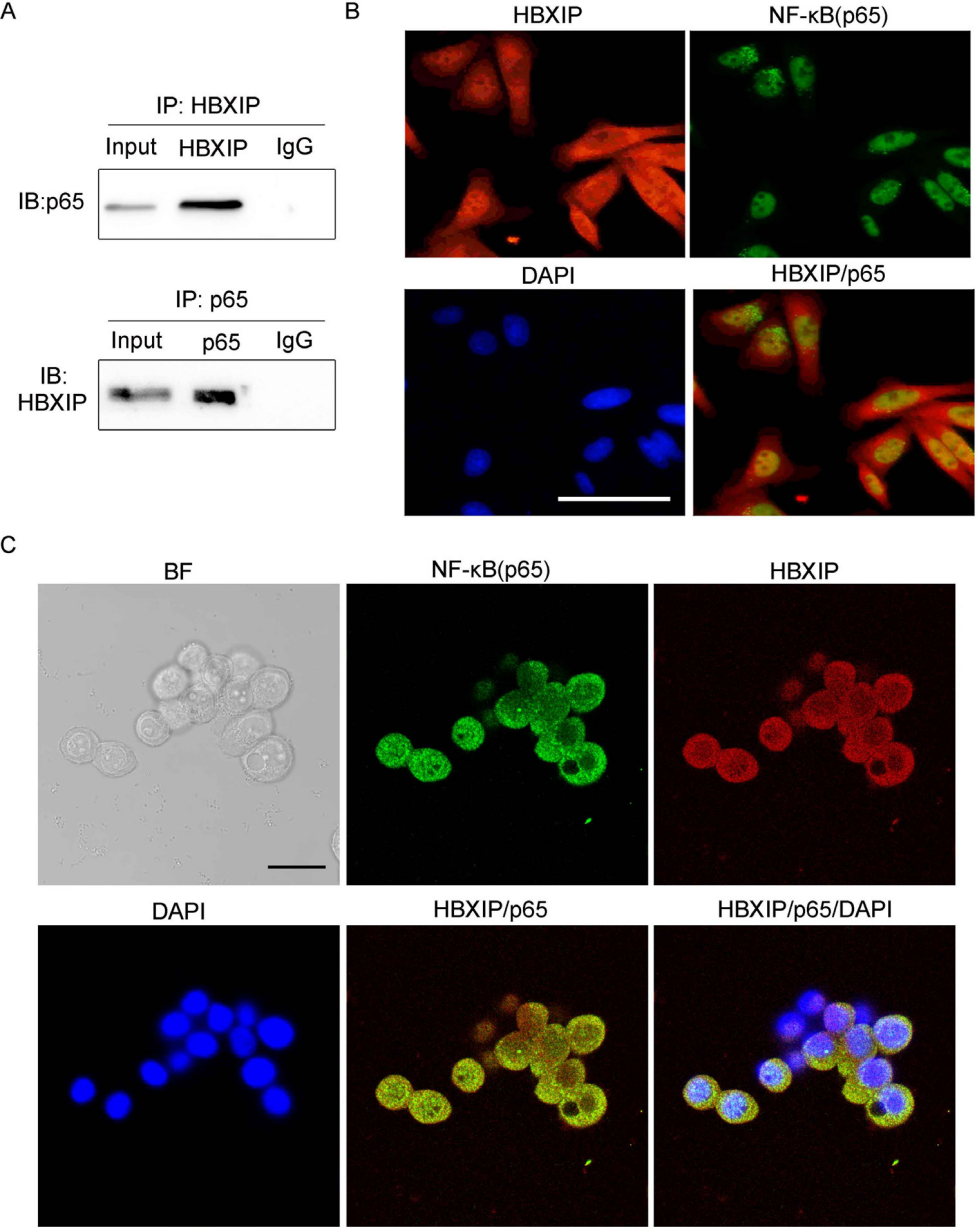
HBXIP binds to NF-κB(p65) in cancer cells (**A**) The interaction between HBXIP and NF-κB was detected by Co-immunoprecipitation (Co-IP) in the HT-29 cancer cells. (**B**) Immunofluorescence and confocal laser scanning microscope (**C**) were performed to detect the association between HBXIP and NF-κB in the HT-29 cells transfected with pCMV-HBXIP (1 μg). The scale bar of immunofluorescence and confocal laser scanning miscroscope is 100 μm and 25 μm, respectively.

To further elucidate the relationship between HBXIP and NF-κB, co-immunoprecipitation (Co-IP) assays were performed and the results displayed that HBXIP and NF-κB could bind to each other in HT-29 cells (Figure 4A). Moreover, immunofluorescence (IF) images showed that the proteins of HBXIP and p65 were mainly located in the cytoplasm and a small amount of HBXIP and p65 proteins co-localized in the nucleus (Figure 4B), the similar results were also confirmed by confocal laser scanning microscope (Figure 4C and Supplementary Figure 3), showing that HBXIP and NF-κB could bind to each other in the nucleus in the cells. Moreover, the western blot results showed that over-expression of HBXIP could elevate the expression of NF-κB/p65 both in the nucleus and cytoplasm (Supplementary Figure 2E). Taken together, we conclude that HBXIP, as a c o-activator of NF-κB, promotes the transcription activity of PPARδ, and enhances its expression in mRNA and protein levels.

### PPARδ regulates the expression of NF-κB in colonic cancer cells

**Figure 5:**
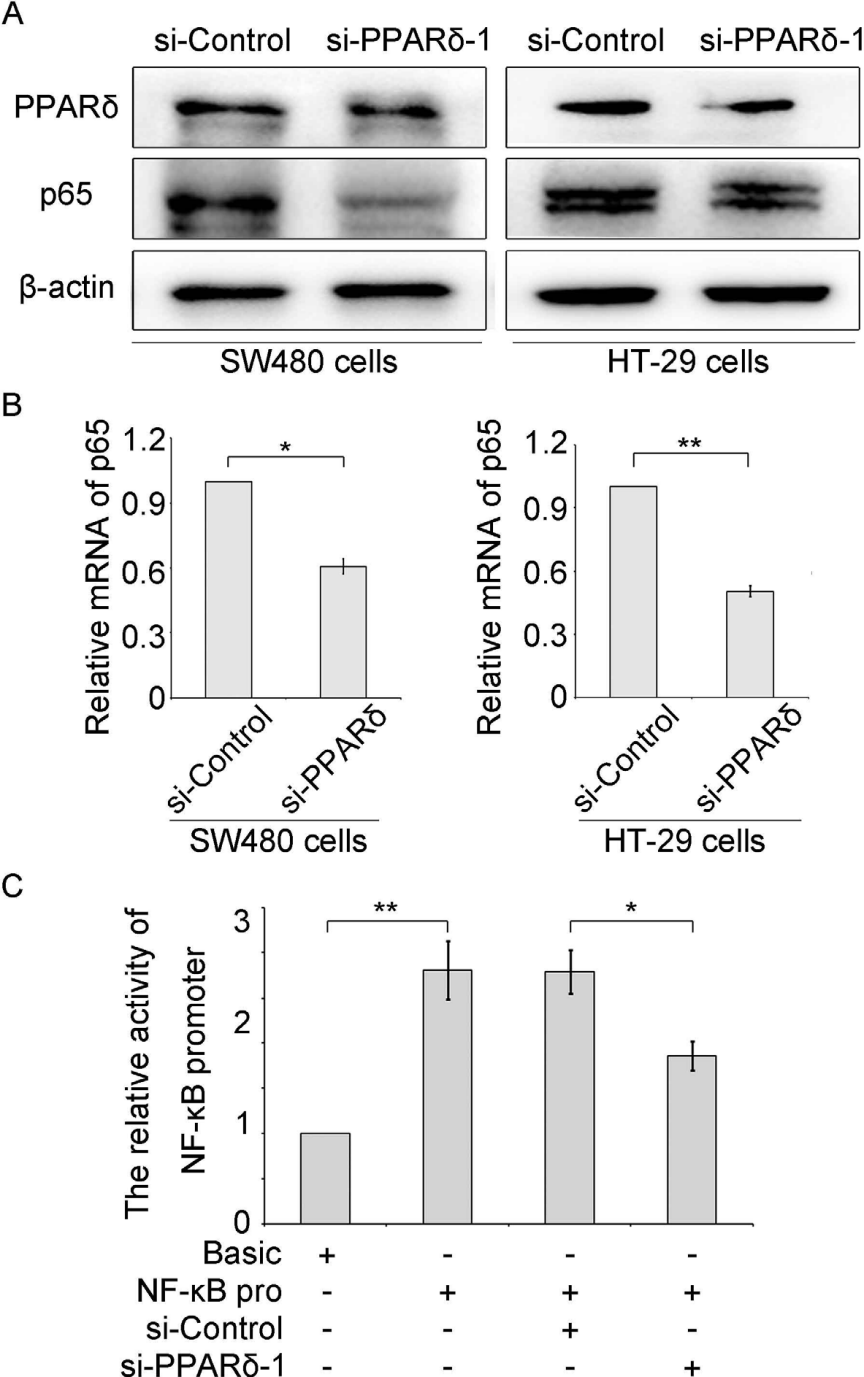
PPARδ enhances the expression of NF-κB via activating its transcription activity (**A**) The protein levels of NF-κB (p65) were detected in SW480 and HT-29 colonic cancer cells transfected with si-PPARδ or si-Control (100 nM), respectively. (**B**) The mRNA levels of NF-κB were measured by quantitative real-time PCR in the cells treated with si-PPARδ or si-Control (100 nM). (**C**) The promoter activity of NF-κB was tested by luciferase reporter gene assays in the colonic cancer HT-29 cells transfected with pGL3-Basic (1 μg), pGL3-NF-κB promoter (1 μg), si-Control (100 nM) and si-PPARδ (100 nM), respectively.

Next, we wondered whether PPARδ could enhance the expression of NF-κB in colonic cancer cells. Interestingly, we observed that PPARδ expression could positively regulate the expression of NF-κB in the cells. Western blot assays were performed in SW480 and HT-29 colonic cancer cells and HEK293T cells. The results indicated that the expression of NF-κB was decreased in SW480 and HT-29 cells transfected with PPARδ siRNA (si-PPARδ) (Figure 5A). The similar results were also confirmed in the HEK293T cells (Supplementary Figure 2D), suggesting that PPARδ could up-regulate the expression of NF-κB in the colonic cancer cells. Then, quantitative real-time PCR (qRT-PCR) were performed and the data showed that the mRNA levels of NF-κB could be remarkably decreased in the cells transfected with si-PPARδ (Figure 5B and Supplementary Figure 2C), suggesting that PPARδ up-regulated the transcription activity of NF-κB. We detected the promoter activity of NF-κB by luciferase reporter gene assays, data showed that the promoter activity of NF-κB could be decreased in the cells treated with si-PPARδ (Figure 5C and Supplementary Figure 4C), suggesting that PPARδ could activate the transcription activity of NF-κB. Taken together, we conclude that PPARδ up-regulated the expression of NF-κB *via* activating its transcription activity in colonic cancer cells.

### PPARδ is required for HBXIP-induced proliferation of colonic cancer cells

**Figure 6:**
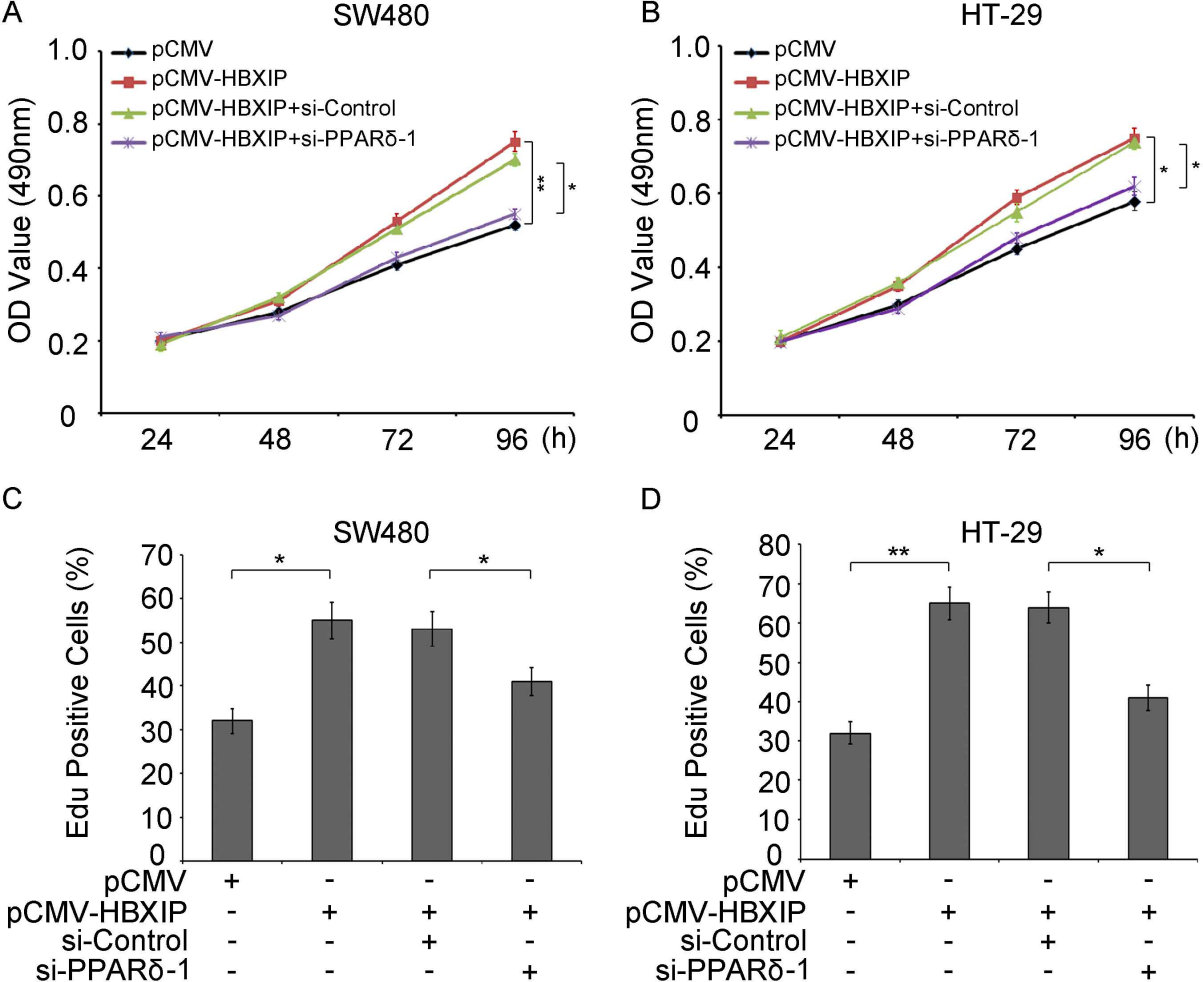
PPARδ is required for HBXIP-induced proliferation of colonic cancer cells (**A**) The effect of PPARδ knowdown on the proliferation of SW480 or HT-29 (**B**) cells transiently cotransfected with pCMV (1 μg), pCMV-HBXIP (1 μg), pCMV-HBXIP (1 μg)/si-Control(100 nM) and pCMV-HBXIP (1 μg)/si-PPARδ (100 nM) were measured by MTT assays (**C**) or detected by EdU assays (**D**), respectively.

Based on the hypothesis that PPARδ is involved in the HBXIP-induced cell proliferation, we performed Methylthiazolyldiphenyl-tetrazolium bromide (MTT) assays in SW480 or HT-29 cells by transfecting with pCMV (0.5 μg), pCMV-HBXIP (0.5 μg), pCMV-HBXIP (0.5 μg)/si-Control (100 nM) or pCMV-HBXIP (0.5 μg)/si-PPARδ (100 nM) respectively. Consistent with the hypothesis, the data clearly indicated that the proliferation ability of SW480 was increased by HBXIP over-expression. However, the knockdown of PPARδ abolished the HBXIP-induced proliferation (Figure 6A, 6B and Supplementary Figure 4A), the similar results were also confirmed by colony formation assays (Supplementary Figure 4B), suggesting that HBXIP promoted the proliferation of colonic cancer cells through activating PPARδ. Moreover, ethynyldeoxyuridine (EdU) assays were performed and the data showed the similar results of MTT assays, that is HBXIP expression enhanced the proliferation of SW480 or HT-29 cells, and the increase was abolished by the addition of si-PPARδ (100 nM) (Figure 6C, 6D and Supplementary Figure 4D). Taken together, these data strongly suggest that HBXIP promoted the proliferation of colonic cancer cells *via* activating PPARδ.

### Association between HBXIP and clinicopathological characteristics

We have found that HBXIP could promote the proliferation of colonic cancer cells. Then we wondered whether the expression levels of HBXIP were associated with specific clinicopathological parameters in colonic cancer patients. 148 patients (Supplementary Table 1) were separated equally into two groups according to the expression levels of HBXIP measured by quantitative real-time PCR (qRT-PCR). We found that HBXIP expression levels were strongly correlated with the clinical stage including lymph node metastasis and advanced TNM stage (Table 1). However, there were no statistically significant associations between HBXIP expression and other clinicopathological data, including Age, Gender, Tumor size and Differentiation. The clinicopathological parameters showed that HBXIP expression was positively associated with the development and progression of colonic carcinoma, which suggests that HBXIP may play important roles in the colonic carcinoma and function as a potential factor for predicting tumor malignancy.

**Table 1:** The relationship between HBXIP expression and clinicopathologic parameters in Colon cancer patients

Clinicopathologic parameters	HBXIP expression^1^		*P*-value
	High (%)	Low (%)	
**All cases**	74	74	
**Age (years)**			0.735
≤65	27 (36.5)	29 (39.2)	
>65	47 (63.5)	45 (60.8)	
**Gender**			0.139
Male	41 (55.4)	32 (43.2)	
Female	33 (44.6)	42 (56.8)	
**Tumor Size (cm)**			0.079
≤5	55 (74.3)	45 (60.8)	
>5	19 (25.7)	29 (39.2)	
**Differentiation**			0.603
low	35 (47.3)	29 (39.2)	
moderate	37 (50)	43 (58.1)	
High	2 (2.7)	2 (2.7)	
**Lymph node metastasis**			0.003**
N0M0	34 (45.9)	52 (70.3)	
N1MO	25 (33.8)	8 (10.8)	
N2M0	9 (12.2)	11 (14.9)	
M1	6 (8.1)	3 (4.0)	
**TNM Stage**			0.003**
I, II	34 (45.9)	52 (70.3)	
III, IV	40 (54.1)	22 (29.7)	

*P < 0.05, **P < 0.01, ***P < 0.001, Chi-squared test *P*-value.

^1^Median relative expression level was used as a cutoff to divide the 148 patients into high HBXIP group (*n* = 74) and low HBXIP group (*n* = 74).

### HBXIP has a positive correlation with PPARδ in clinical colonic carcimoma tissues

**Figure 7:**
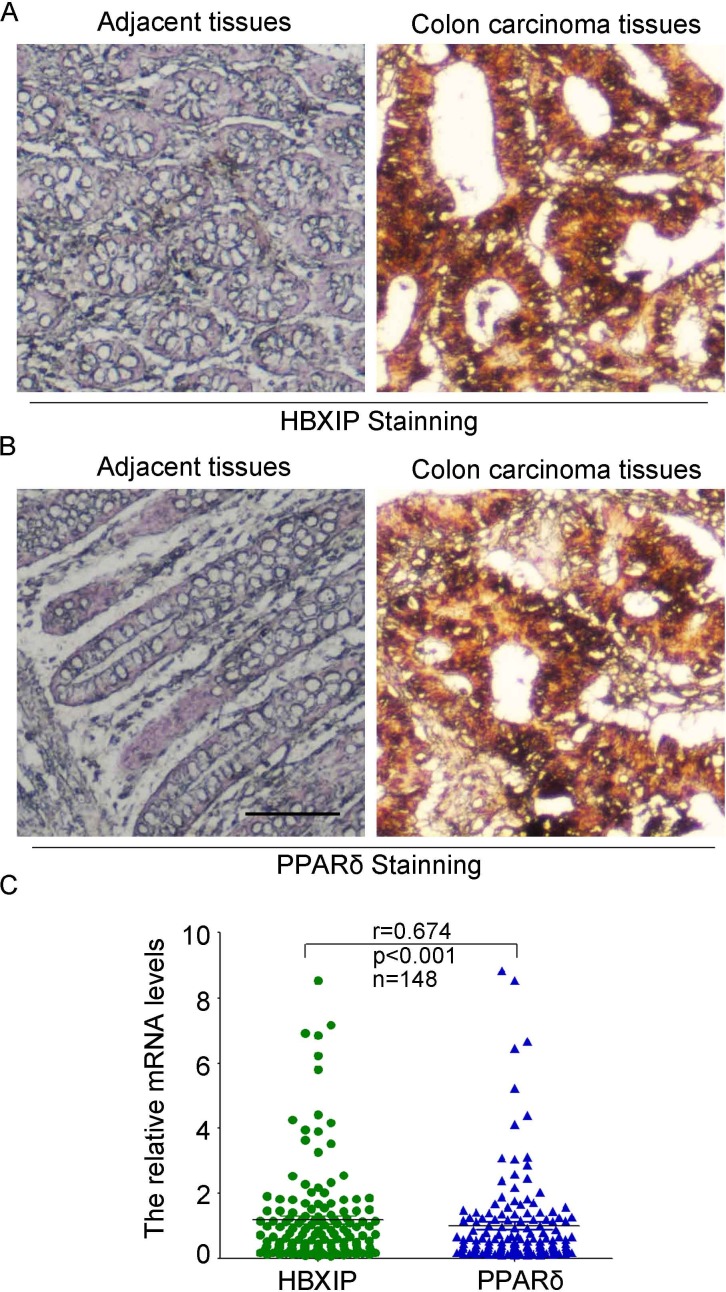
HBXIP has a significant positive correlation with PPARδ in clinical colonic carcinoma tissues (**A**) The expression levels of HBXIP and PPARδ (**B**) were detected in the colonic carcinoma tissues and adjacent normal tissues by immunohistochemical staining. The scale bar is 100 μm. (**C**) The correlation of mRNA levels of HBXIP and PPARδ was examined by quantitative real-time PCR (qRT-PCR) in 148 colonic carcinoma paraffin-embedded specimen (*p* < 0.001, *r* = 0.674, Pearson’s correlation).

Literatures reported that PPARδ, a ligand-inducible transcription factor, is highly expressed in colonic cells and involved in colonic tumorigenesis. Thus, we wondered whether the expression of HBXIP was associated with that of PPARδ in the clinical colonic cancer patients. We then tested the expression levels of HBXIP (Figure 7A) and PPARδ (Figure 7B) of 83 cases of colonic paraffin-embedded specimen and 12 cases of normal colonic tissues by immunohistochemical assays (Supplementary Table 3), the results demonstrated that Both HBXIP and PPARδ were highly expressed in the colonic tumor tissues and showed a positive correlation between HBXIP and PPARδ expression in the carcinoma tissues, which suggests that HBXIP had played important roles in the progression of colonic carcinoma cells. Moreover, quantitative real-time PCR were performed to test the expression levels of HBXIP and PPARδ of the colonic carcinoma tissues from 148 colonic carcinoma paraffin-embedded specimen. The data demonstrated that the expression levels of PPARδ had a significant positive correlation with those of HBXIP in colonic carcinoma tissues (*p* < 0.001 Wilcoxon signed-rank, Figure 7C). Taken together, we conclude that the expression levels of HBXIP are positively associated with those of PPARδ in colonic carcinoma tissues, and the HBXIP/ PPARδ signaling may have important roles in the colonic tumorigenesis.

## DISCUSSION

HBXIP (LAMTOR5) plays an important role in the promotion of proliferation of breast cancer cells [4–6]. Research reported that HBXIP showed a high expression in the colonic cancer cells (https://www.proteinatlas.org/ENSG00000134248-LAMTOR5/tissue) and we also found the different expression levels of HBXIP in the colonic cancer cells by RT-PCR assays (Supplementary Figure 1A). Previous report indicated that HBXIP functioned in the cytoplasm [25], which is consistent with our immunofluorescent results indicated that HBXIP mainly localized in the cytoplasm, while a small amount of HBXIP proteins located in the nuclues (Figure 4H), suggesting HBXIP may have some functions in the nucleus, which is consistent with our previous studies [4, 6]. However, the mechanism by which HBXIP enhances the proliferation of colonic cancer cells is still unclear. Many studies demonstrated that PPARδ, a ligand-inducible transcription factor, plays a critical role in regulating cancer progression, and PPARδ-related tumorigenesis was first identified in colorectal cancer [26]. PPARδ is also highly expressed in colonic epithelial cells and closely linked to colonic carcinogenesis [20]. Thus, we are interested in whether PPARδ is involved in HBXIP-enhanced proliferation of colonic cancer cells. In our study, we investigate how HBXIP functions with PPARδ in promotion of colonic cancer cells.

Many studies indicated that HBXIP promotes the proliferation of breast cancer cells by different signal pathways [27, 28], and HBXIP expression occurs in nearly all tissues [3]. PPARδ is also found high expression in colonic epithelial cells and involved in colonic carcinogenesis. Thus, we first tested whether HBXIP is associated with PPARδ in colonic cancer cells, we found that HBXIP could up-regulate the expression of PPARδ in mRNA and protein levels in SW480 and HT-29 colonic cancer cells, respectively. In order to explore the underlying mechanism by which HBXIP regulates PPARδ expression, and according to our previous research results that is HBXIP functioned as a co-activator of transcription factors to activate the transcription activity of target genes [4, 6, 27]. ChIP assay results indicated that HBXIP could occupy on the PPARδ promoter, and luciferase report gene data showed that HBXIP could activate the transcription activity of PPARδ. We next reconstructed various length of PPARδ promoter regions and performed the luciferase reporter gene assays, we found the core region of PPARδ promoter, which showed the maximum activity among these different PPARδ promoter regions. Our previous study demonstrated that HBXIP could not bind to DNA sequence directly [4]. Next, we predicted the possible transcription factors of PPARδ promoter using the GPMiner (http://gpminer.mbc.nctu.edu.tw/). NF-κB transcription factor was screened out and previous literature reported that NF-κB signaling was involved in carcinogenesis [24]. Luciferase reporter analysis results validate that NF-κB plays critical roles in regulating the transcription activity of PPARδ. Co-IP, immunofluorescence and confocal laser scanning microscope assays displayed that HBXIP and NF-κB could bind to each other in the cells, indicating that HBXIP up-regulates the expression of PPARδ *via* NF-κB. Thus, HBXIP functioned as a co-activator of NF-κB activates PPARδ transcription. These findings are consistent with our previous report that HBXIP may function as a co-activator [4, 29]. Previous studies indicated that PPARδ negatively regulated the transcription of NF-κB, such as in inflammation [30] or in diabetic nephropathy [31]. Interestingly, we observed that PPARδ up-regulated the expression of NF-κB through activating its transcription activity. One possible explanation is that the mechanisms by which PPARδ regulates NF-κB in inflammation might be different from that in colonic cancer. Thus, HBXIP accelerated the expression of PPARδ/NF-κB feedback loop in the cells. In function, MTT, EdU and colony formation data demonstrated that HBXIP enhanced the proliferation of colonic cancer cells in PPARδ-dependent manner. In clinical colonic cancer samples, we detected the relationship between HBXIP and clinicopathologic parameters in colonic cancer patients. Interestingly, we found that HBXIP is positively correlated with the lymph node metastasis and advanced TNM stage, suggesting HBXIP expression is positively correlated with the development and progression of colonic carcinoma.

In summary, we show a model that the oncoprotein HBXIP promotes the proliferation of colonic cancer cells *via* activating PPARδ, in which HBXIP, functioned as a co-activator of NF-κB, up-regulated the expression of PPARδ, and the PPARδ conversely up-regulated the expression of NF-κB through activating its transcription activity (Supplementary Figure 5). The HBXIP/PPARδ/NF-κB signaling may be a critical key driver in the development and progression of colonic carcinogenesis. Our findings provide new insights into the mechanism by which HBXIP promoted the proliferation of colonic cancer cells. Additionally, HBXIP may serve as a potential biomarker or drug target for diagnosis and treatment for colonic cancer patients.

## MATERIALS AND METHODS

### Clinical specimen collection

A total of 148 colonic carcinoma paraffin-embedded specimen were obtained from patients who underwent surgical resection at the Changzhou Wujin People’s Hospital. This study was approved by the Human Ethics Committee board of Changzhou Wujin People’s Hospital and informed consent was obtained from patients prior to the research commencing with no patients receiving preoperative treatments. The information of patients with colonic cancer is presented in Supplementary Table 1, which was tested by quantitative real-time PCR, and 83 cases from the 148 colonic carcinoma paraffin-embedded specimens were detected by immunohistochemistry assays. The documented clinicopathological characteristics were: Age, Gender, Tumor size, Differentiation, Lymph node metastasis and TNM stage.

### Cell culture and treatment

Colonic cancer cell line SW480 and HT-29 cells and HEK293T cells were cultured in Dulbecco’s Modified Eagle Medium, DMEM (Gibco by life technologies), with 10% Fetal Bovine Serum (FBS) supplemented with 100 U/ml penicillin, 100 U/ml streptomycin and 1% glutamine at 37°C with 5% CO2. Transfections were performed by transfecting plasmids pCMV-Tag2B, pCMV-HBXIP or pCMV-p65 into colonic cancer SW480 or HT-29 cells with TurboFect Transfection Reagent (Thermo Scientific). Cells were collected and seeded in 6-well, 24-well or 96-well plates for 24 hr and then were transfected with plasmids or siRNAs. All transfections were performed with TurboFect Transfection Reagent according to (Thermo Scientific) manufacturer’s instructions. The siRNAs used in our study were as follows: negative control siRNA (si-Control), HBXIP siRNA (si-HBXIP), p65 siRNA (si-p65) and PPARδ siRNA (si-PPARδ) (Shanghai GenePharma Co., Ltd).

### RT-PCR, qRT-PCR and Western blotting assays

The total RNA of paraffin-embedded specimens was extracted by using the Recover All Total Nucleic Acid Isolation Optimized for FFPE Sample Kit (Ambion Inc., Texas, USA) following the manufacturer’s protocol [32]. And the total RNA from the cells was extracted using Trizol (Invitrogen) according to the manufacturer’s protocol. For HBXIP, PPARD and P65 mRNA level detection, the total RNA from colonic cancer SW480, HT-29 cells or from the paraffin-embedded carcinoma specimens were directly reverse transcribed. The qRT-PCR was performed as described in the method of SYBR Premix Ex Tag Kit (TaKaRa, Japan). All primers used in this study are listed in Supplementary Table 2. Western blotting was performed as previously described [33]. The primary antibodies used in this study were rabbit polyclonal anti-HBXIP, anti-PPARδ, anti-p65 (BBI, Sangon Biotech) and anti-β-actin (Santa Cruz Biotechnology, Santa Cruz, CA).

### Immunohistochemistry

Immunohistochemistry assay was performed as described previously [33]. The slides provided by Pathology of Changzhou Wujin People’s Hospital were incubated with rabbit anti-HBXIP (or rabbit anti- PPARδ) antibody at 4°C for overnight. After incubation at room temperature for 30 min with biotinylated secondary antibody, the slides were stained with the 3-amino-9-ethylcarbazole according to the protocol of AEC substrate staining Kit (Solarbio).

### Plasmids construction

The 5′-flanking region (from –1491 to –239 nt) of PPARδ gene was cloned into the KpnI/MluI site of promoterless luciferase construct pGL3-Basic vector, –1491/–239 (pGL3-1253) (Promega). Then, the recombinant plasmid pGL3-1253 was used as the template to clone the rest of the truncated promoter region, −1296/−239 (pGL3-1058), −1079/−239 (pGL3-841), −835/−239 (pGL3-597), −599/−239 (pGL3-361) and −389/−239 (pGL3-151) of PPARδ with the same KpnI/MluI site of the pGL3-Basic vector, respectively. All primers are listed in Supplementary Table 2.

### Co-immunoprecipitation (Co-IP) assay

The co-immunoprecipitation protocol was described in detail on our previously published paper [4]. SW480 cells (2 × 10^6^ ) were harvested and lysed in a lysis buffer (50 mM Tris-HCl pH 7.5, 150 mM NaCl, 1 mM EDTA, 0.3% Triton X-100, 1 mM protease inhibitor PMSF). The lysates were incubated with antibodies at 4°C overnight, next added the pre-washed protein A-conjugated agarose beads at 4°C for 2 h. The precipitates were washed eight times with ice-cold lysis buffer, resuspended in the PBS and resolved by SDS-PAGE followed by western blotting.

### Luciferase reporter gene assay

Adherent cells (SW480 and HT-29 cells) were seeded into 24-well plates and, respectively, transfected with the constructs containing different length fragments of PPARδ promoter or pGL3-Basic as a negative control, with the pRL-TK plasmid (Promega, Madison, WI) which was used as internal normalization. Cell extracts were harvested after 36 hours and lysed using lysis buffer (Promega). Luciferase reporter gene assay was measured using the GLOMA MULTI DETECTION SYSTEM (Promega) as previously described [4]. All experiments were performed at least three times.

### Chromatin immunoprecipitation (ChIP) assays

The chromatin immunoprecipitation assays were performed by the published methods [34], and the detailed procedures were carried out according to the protocol of the EZ-Magna ChIP™ A–Chromatin Immunoprecipitation Kit (Merck Millipore). Protein-DNA complexes were immunoprecipitated with HBXIP antibodies and with mouse IgG as a negative control antibody. DNA collected by these antibodies was subjected to PCR analysis, followed by sequencing. Amplification of soluble chromatin prior to immunoprecipitation was used as an input control.

### Analysis of cell proliferation

SW480 and HT-29 cells were seeded respectively onto 96 well plates (800 cells/well) for 24 h before transfection and the 3-(4,5-dimethylthiazol2-yl) 22, 5-diphenyltetrazolium bromide (MTT) (Sigma) assays [4] were used to assess cell proliferation every day from the 24 h until the 96 h after transfection. In addition, 5-ethynyl-2 0 -deoxyuridine (EdU) incorporation assay was carried out to assess cell proliferation using the Cell-Light TM EdU imaging detecting kit according to the manufacturer’s instructions (RiboBio, China) as well. For the colony formation assays, approximately 1000 SW480 transfected cells or 3000 HT29 transfected cells were placed in six-well plates after 36 h transfection and maintained in complete medium for 10 days, respectively. The colonies were fixed by methanol for 20 min then stained with methylene blue for 30 min, washed with water and take pictures as previous described [4].

### Statistical analysis

Each experiment was repeated at least three times. Statistical significance was assessed by comparing mean values (±SD) using a Student’s *t*-test for independent groups and was assumed for *p* < 0.05 (*), *p* < 0.01 (**) and *p* < 0.001 (***). Correlation between expression levels of HBXIP and PPARδ in tumor tissues was explored using Pearson’s correlation coefficient by the Statistical Analysis System (GraphPad Prism 6, GraphPad Software Inc, CA, USA)

## SUPPLEMENTARY MATERIALS FIGURES AND TABLES









## References

[1] Ferlay J, Soerjomataram I, Dikshit R, Eser S, Mathers C, Rebelo M, Parkin DM, Forman D, Bray F (2015). Cancer incidence and mortality worldwide: sources, methods and major patterns in GLOBOCAN 2012. Int J Cancer.

[2] Bar-Peled L, Schweitzer LD, Zoncu R, Sabatini DM (2012). Ragulator is a GEF for the rag GTPases that signal amino acid levels to mTORC1. Cell.

[3] Melegari M, Scaglioni PP, Wands JR (1998). Cloning and characterization of a novel hepatitis B virus x binding protein that inhibits viral replication. J Virol.

[4] Liu Q, Bai X, Li H, Zhang Y, Zhao Y, Zhang X, Ye L (2013). The oncoprotein HBXIP upregulates Lin28B via activating TF II D to promote proliferation of breast cancer cells. Int J Canc Prev.

[5] Wang Y, Cai X, Zhang S, Cui M, Liu F, Sun B, Zhang W, Zhang X, Ye L (2017). HBXIP up-regulates ACSL1 through activating transcriptional factor Sp1 in breast cancer. Biochem Biophys Res Commun.

[6] Yue L, Li L, Liu F, Hu N, Zhang W, Bai X, Li Y, Zhang Y, Fu L, Zhang X, Ye L (2013). The oncoprotein HBXIP activates transcriptional coregulatory protein LMO4 via Sp1 to promote proliferation of breast cancer cells. Carcinogenesis.

[7] Hall MG, Quignodon L, Desvergne B (2008). Peroxisome Proliferator-Activated Receptor beta/delta in the Brain: Facts and Hypothesis. PPAR Res.

[8] Gou Q, Gong X, Jin J, Shi J, Hou Y (2017). Peroxisome proliferator-activated receptors (PPARs) are potential drug targets for cancer therapy. Oncotarget.

[9] Rosenson RS, Wright RS, Farkouh M, Plutzky J (2012). Modulating peroxisome proliferator-activated receptors for therapeutic benefit? Biology, clinical experience, and future prospects. Am Heart J.

[10] Heck BE, Park JJ, Makani V, Kim EC, Kim DH (2017). PPAR-delta agonist with mesenchymal stem cells induces type II collagen-producing chondrocytes in human arthritic synovial fluid. Cell Transplant.

[11] Abd El-Haleim EA, Bahgat AK, Saleh S (2016). Effects of combined PPAR-gamma and PPAR-alpha agonist therapy on fructose induced NASH in rats: Modulation of gene expression. Eur J Pharmacol.

[12] Hall MG, Quignodon L, Desvergne B (2008). Peroxisome Proliferator-Activated Receptor beta/delta in the Brain: Facts and Hypothesis. PPAR research.

[13] Crunkhorn S (2016). Huntington disease: Boosting PPARdelta blocks neurodegeneration. Nature reviews Drug discovery.

[14] Zhang W, Xu Y, Xu Q, Shi H, Shi J, Hou Y (2017). PPARdelta promotes tumor progression via activation of Glut1 and SLC1-A5 transcription. Carcinogenesis.

[15] Angione AR, Jiang C, Pan D, Wang YX, Kuang S (2011). PPARdelta regulates satellite cell proliferation and skeletal muscle regeneration. Skeletal muscle.

[16] Ji MJ, Yu XB, Mei ZL, An YQ, Tang SS, Hu M, Long Y, Miao MX, Hu QH, Sun HB, Kong LY, Hong H (2015). Hippocampal PPARdelta Overexpression or Activation Represses Stress-Induced Depressive Behaviors and Enhances Neurogenesis. Int J Neuropsychopharmacol.

[17] Gupta RA, Tan J, Krause WF, Geraci MW, Willson TM, Dey SK, DuBois RN (2000). Prostacyclin-mediated activation of peroxisome proliferator-activated receptor delta in colorectal cancer. Proc Natl Acad Sci U S A.

[18] Zuo X, Peng Z, Moussalli MJ, Morris JS, Broaddus RR, Fischer SM, Shureiqi I (2009). Targeted genetic disruption of peroxisome proliferator-activated receptor-delta and colonic tumorigenesis. J Natl Cancer Inst Monogr.

[19] He TC, Chan TA, Vogelstein B, Kinzler KW (1999). PPARdelta is an APC-regulated target of nonsteroidal anti-inflammatory drugs. Cell.

[20] Jeong E, Koo JE, Yeon SH, Kwak MK, Hwang DH, Lee JY (2014). PPARdelta deficiency disrupts hypoxia-mediated tumorigenic potential of colon cancer cells. Mol Carcinog.

[21] Wang D, DuBois RN (2014). PPARdelta and PGE2 signaling pathways communicate and connect inflammation to colorectal cancer. Inflamm Cell Signal.

[22] Wang Y, Sun J, Li N, Che S, Jin T, Liu S, Lin Z (2017). HBXIP overexpression is correlated with the clinical features and survival outcome of ovarian cancer. J Ovarian Res.

[23] Wang Y, Fang R, Cui M, Zhang W, Bai X, Wang H, Liu B, Zhang X, Ye L (2017). The oncoprotein HBXIP up-regulates YAP through activation of transcription factor c-Myb to promote growth of liver cancer. Cancer Lett.

[24] Shen HM, Tergaonkar V (2009). NFkappaB signaling in carcinogenesis and as a potential molecular target for cancer therapy. Apoptosis.

[25] Marusawa H, Matsuzawa S, Welsh K, Zou H, Armstrong R, Tamm I, Reed JC (2003). HBXIP functions as a cofactor of survivin in apoptosis suppression. EMBO J.

[26] You M, Yuan S, Shi J, Hou Y (2015). PPARdelta signaling regulates colorectal cancer. Curr Pharm Des.

[27] Liu S, Li L, Zhang Y, Zhang Y, Zhao Y, You X, Lin Z, Zhang X, Ye L (2012). The oncoprotein HBXIP uses two pathways to up-regulate S100A4 in promotion of growth and migration of breast cancer cells. J Biol Chem.

[28] Liu F, Zhang W, You X, Liu Y, Li Y, Wang Z, Wang Y, Zhang X, Ye L (2015). The oncoprotein HBXIP promotes glucose metabolism reprogramming via downregulating SCO2 and PDHA1 in breast cancer. Oncotarget.

[29] Liu F, You X, Wang Y, Liu Q, Liu Y, Zhang S, Chen L, Zhang X, Ye L (2014). The oncoprotein HBXIP enhances angiogenesis and growth of breast cancer through modulating FGF8 and VEGF. Carcinogenesis.

[30] Schnegg CI, Kooshki M, Hsu FC, Sui G, Robbins ME (2012). PPARdelta prevents radiation-induced proinflammatory responses in microglia via transrepression of NF-kappaB and inhibition of the PKCalpha/MEK1/2/ERK1/2/AP-1 pathway. Free Radic Biol Med.

[31] Liang YJ, Jian JH, Liu YC, Juang SJ, Shyu KG, Lai LP, Wang BW, Leu JG (2010). Advanced glycation end products-induced apoptosis attenuated by PPARdelta activation and epigallocatechin gallate through NF-kappaB pathway in human embryonic kidney cells and human mesangial cells. Diabetes Metab Res Rev.

[32] Gouveia GR, Ferreira SC, Ferreira JE, Siqueira SA, Pereira J (2014). Comparison of two methods of RNA extraction from formalin-fixed paraffin-embedded tissue specimens. Biomed Res Int.

[33] Gao J, Liu Q, Xu Y, Gong X, Zhang R, Zhou C, Su Z, Jin J, Shi H, Shi J, Hou Y (2015). PPARalpha induces cell apoptosis by destructing Bcl2. Oncotarget.

[34] Nelson JD, Denisenko O, Bomsztyk K (2006). Protocol for the fast chromatin immunoprecipitation (ChIP) method. Nat Protoc.

