# Supplementary material for: HBXIP activates the PPARδ/NF-κB feedback loop resulting in cell proliferation
Source: Oncotarget. 2017 Dec 8;9(1):404–17. doi: 10.18632/oncotarget.23057 (PMC5787476; doi:10.18632/oncotarget.23057)
Supplement: Supplementary file 1 [file oncotarget-09-404-s001.pdf]

HBXIP activates the PPARδ/NF-κB feedback loop resulting in cell proliferation

SUPPLEMENTARY MATERIALS

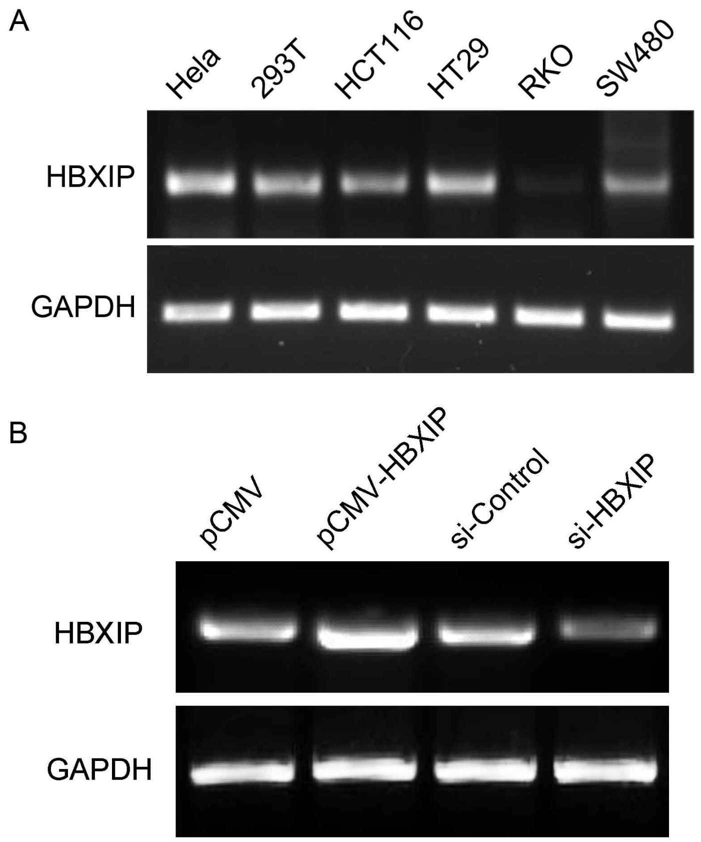

Supplementary Figure 1: The mRNA levels of HBXIP in colonic cell lines. (**A**) The mRNA levels of HBXIP in different colonic cell lines. (**B**) The mRNA levels of HBXIP were detected by RT-PCR experiments in the colonic cancer SW480 cells treated with pCMV-Tag2B (1 μg), pCMV-HBXIP (1 μg), si-Control (100 nM) and si-HBXIP (100 nM), respectively. GAPDH served as an internal reference.

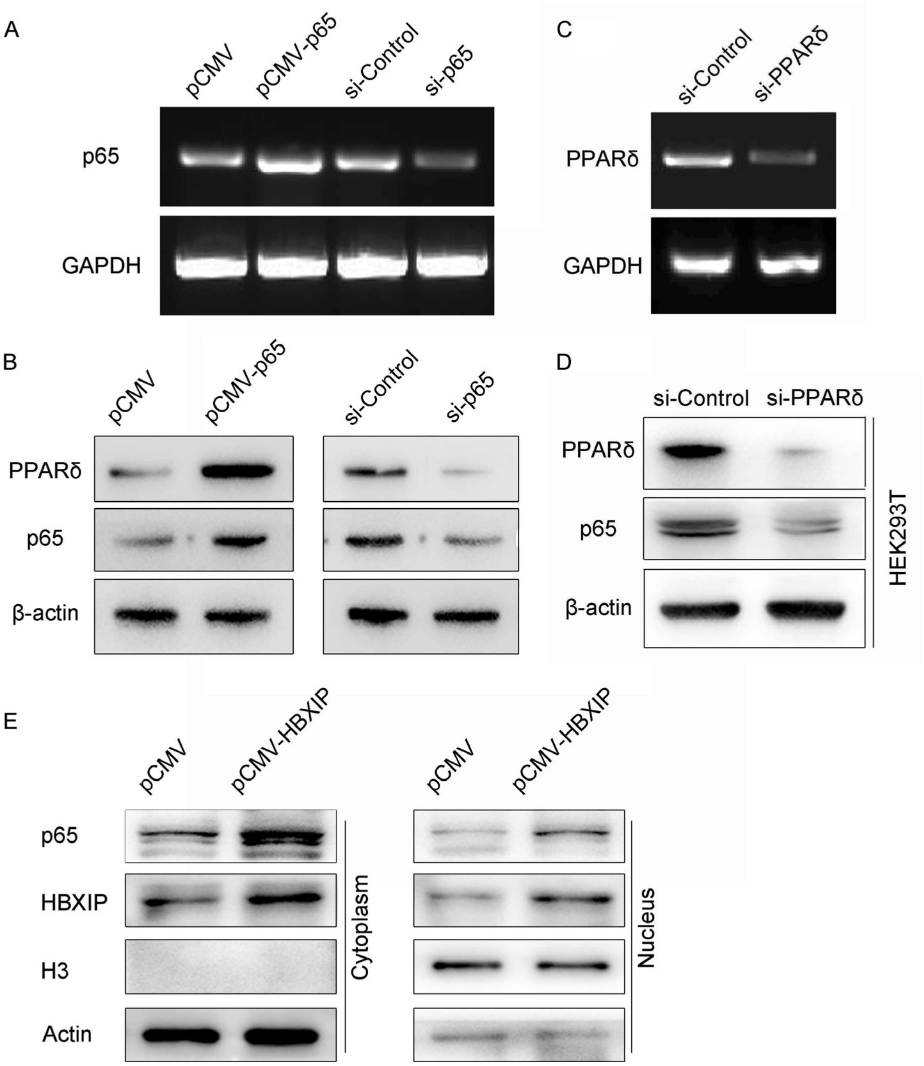

Supplementary Figure 2: The positive feedback regulation between NF-κB/p65 and PPARδ. (**A**) The mRNA and protein levels of NF-κB (p65) were measured by RT-PCR and Western blot assays (**B**) in the colonic cancer HT-29 cells transfected with pCMV-Tag2B (0.5 μg), pCMV-p65 (0.5 μg), si-Control (100 nM) and si-p65 (100 nM), respectively. (**C**) The mRNA and protein levels of PPARδ were measured by RT-PCR and Western blotting (**D**) in the colonic cancer HT-29 cells transfected with si-Control (100 nM) and si-p65 (100 nM), respectively. GAPDH and β-actin served as an internal reference. (**E**) The protein levels of p65 and HBXIP extracted respectively from cytoplasm and nucleus were tested by Western blotting in the cells transfected with pCMV-Tag2B (0.5 μg) and pCMV-HBXIP (0.5 μg) respectively.

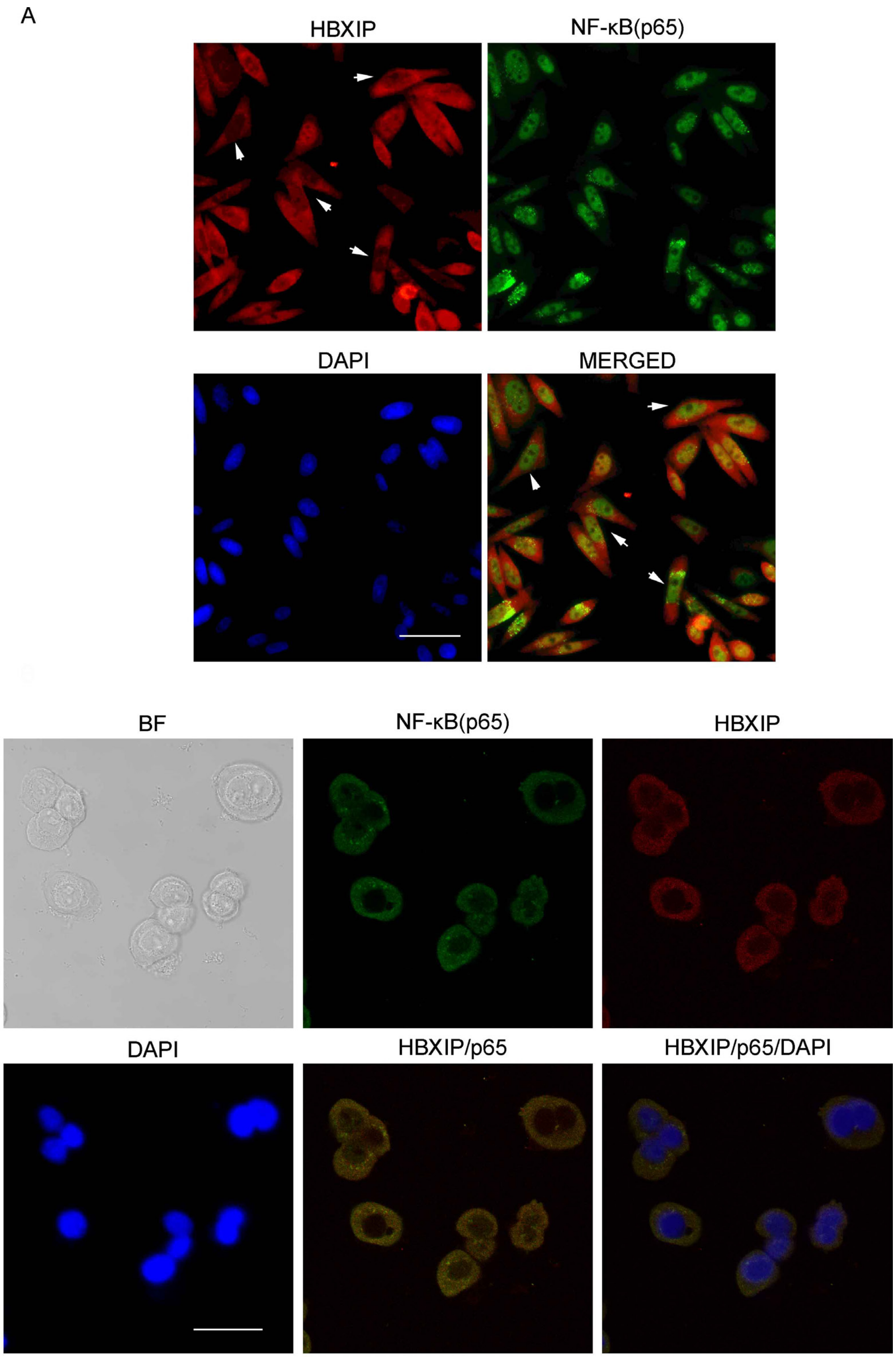

Supplementary Figure 3: The colocalization between HBXIP and NF-κB/p65. The colocalization between endogeneous HBIXP and NF-κB/p65 were detected by immunofluorescence (**A**) and confocal laser scanning microscope (**B**) in the colonic cancer HT-29 cells, respectively. The scale bar of immunofluorescence confocal assay is 100 μm and 25 μm, respectively.

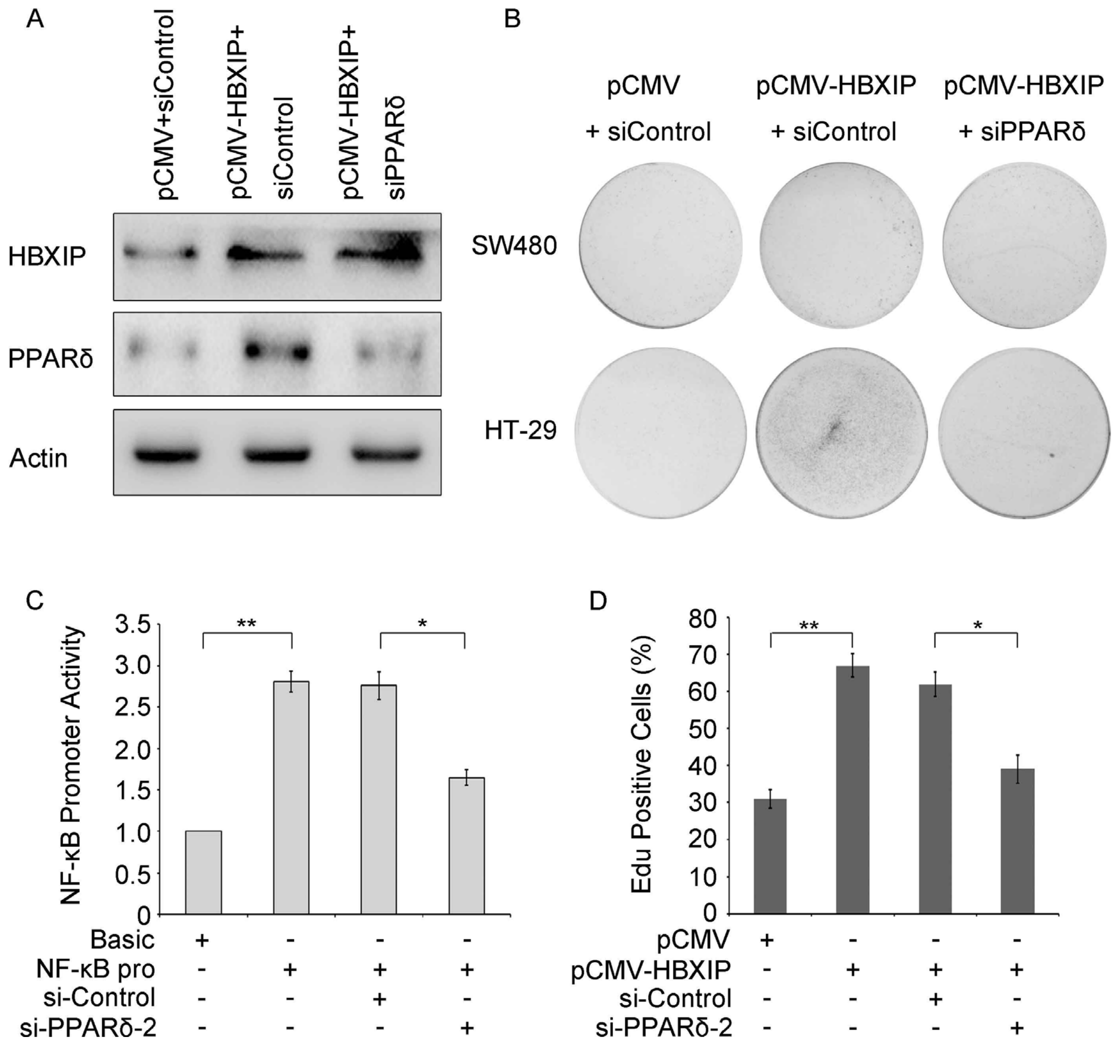

Supplementary Figure 4: HBXIP promoted the proliferation of colonic cancer cells via activating PPARδ. (**A**) The over-expression and interruption efficiency were detected by western blotting. (**B**) HBXIP promoting the proliferation of colonic cancer cells via PPARδ was tested by colony formation. (**C**) Another PPARδ siRNA (si-PPARδ-2) was used to measure the activity of NF-κB/p65 promoter by the Luciferase reporter assays and detected the proliferation ratio (**D**) of the cancer cells transfected with pCMV-Tag2B (0.5 μg), pCMV-HBXIP (0.5 μg), si-Control (100 nM) and si-PPARδ-2 (100 nM) by Edu assays, respectively.

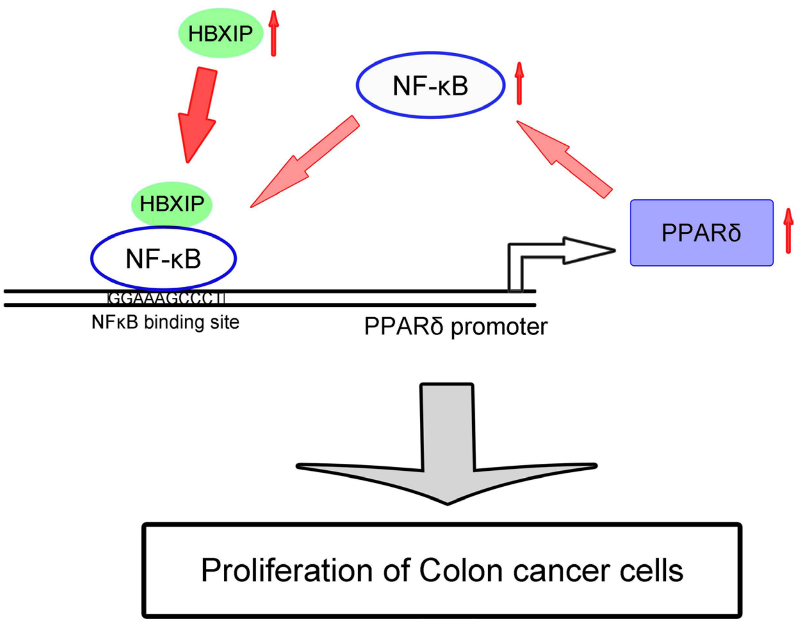

Supplementary Figure 5: The model displays the oncoprotein HBXIP activating the expression of feedback loop of PPARδ/NF-κB to promote the proliferation of colonic cancer cells. The HBXIP binds to NF-κB in the nucleus to activate the PPARδ promoter. Conversely, PPARδ enhances the expression of NF-κB *via* activating its transcription activity. Thus, HBXIP accelerated the positive feedback loop of PPARδ/NF-κB, which resulted in the fast growth of colonic cancer cells.

Supplementary Table 1: Clinical characteristics of colon carcinoma paraffin samples. See Supplementary_Table_1

Supplementary Table 2: The primers used in this study. See Supplementary_Table_2

Supplementary Table 3: Specimens used in immunohistochemistry assays. See Supplementary_Table_3
